# Elevated plasma free fatty acids increase cardiovascular risk by inducing plasma biomarkers of endothelial activation, myeloperoxidase and PAI-1 in healthy subjects

**DOI:** 10.1186/1475-2840-9-9

**Published:** 2010-02-16

**Authors:** Manoj Mathew, Eric Tay, Kenneth Cusi

**Affiliations:** 1 Diabetes Division, Department of Medicine, The University of Texas Health Science Center at San Antonio, Texas-78229, USA; 2 Audie L Murphy Veterans Administration Medical Center, San Antonio, Texas-78229, USA

## Abstract

**Background:**

CVD in obesity and T2DM are associated with endothelial activation, elevated plasma vascular inflammation markers and a prothrombotic state. We examined the contribution of FFA to these abnormalities following a 48-hour *physiological *increase in plasma FFA to levels of obesity and diabetes in a group of healthy subjects.

**Methods:**

40 non-diabetic subjects (age = 38 ± 3 yr, BMI = 28 ± 1 kg/m^2^, FPG = 95 ± 1 mg/dl, HbA_1c _= 5.3 ± 0.1%) were admitted twice and received a 48-hour infusion of normal saline or low-dose lipid. Plasma was drawn for intracellular (ICAM-1) and vascular (VCAM-1) adhesion molecules-1, E-selectin (sE-S), myeloperoxidase (MPO) and total plasminogen inhibitor-1 (tPAI-1). Insulin sensitivity was measured by a hyperglycemic clamp (M/I).

**Results:**

Lipid infusion increased plasma FFA to levels observed in obesity and T2DM and reduced insulin sensitivity by 27% (p = 0.01). Elevated plasma FFA increased plasma markers of endothelial activation ICAM-1 (138 ± 10 vs. 186 ± 25 ng/ml), VCAM-1 (1066 ± 67 vs. 1204 ± 65 ng/ml) and sE-S (20 ± 1 vs. 24 ± 1 ng/ml) between 13-35% and by ≥ 2-fold plasma levels of myeloperoxidase (7.5 ± 0.9 to 15 ± 25 ng/ml), an inflammatory marker of future CVD, and tPAI-1 (9.7 ± 0.6 to 22.5 ± 1.5 ng/ml), an indicator of a prothrombotic state (all p ≤ 0.01). The FFA-induced increase was independent from the degree of adiposity, being of similar magnitude in lean, overweight and obese subjects.

**Conclusions:**

An increase in plasma FFA within the physiological range observed in obesity and T2DM induces markers of endothelial activation, vascular inflammation and thrombosis in healthy subjects. This suggests that even transient (48-hour) and modest increases in plasma FFA may initiate early vascular abnormalities that promote atherosclerosis and CVD.

## Background

Endothelial cells (ECs) play a key role in the transport of metabolic substrates and cells between the blood and the interstitial space, including a complex signalling system that regulates innate and immune responses of the vascular bed [1,2]. Transendothelial migration of leukocytes is regulated by soluble cell adhesion molecules such as intercellular adhesion molecule-1 (ICAM-1), vascular adhesion molecule-1 (VCAM-1) and E-selectin. Their expression is increased as ECs are activated by proinflammatory stimuli such as bacterial endotoxins, Il-1b or TNF-α, CRP, oxidized LDL or hemodynamic forces related to blood flow [3,4]. EC activation involves the NF-Kβ and other intracellular inflammatory pathways and play a key role in the early development of the inflammatory response in atherosclerosis [3-7]. Atherosclerosis in obesity, metabolic syndrome and T2DM is initiated by damage/activation of the endothelium [2,4,8]. Plasma measurement of cell adhesion molecules are accepted markers of endothelial dysfunction and vascular disease [9-16]. Endothelial activation has procoagulant consequences that can be measured as a change in the balance of tissue plasminogen activator and its endogenous inhibitor, tissue plasminogen activation inhibitor-1 (tPAI-1 or PAI-1) [17]. Plasminogen activator inhibitor 1 is the primary physiological inhibitor of tissue-type plasminogen activator and urokinase-like plasminogen activator and inhibits both fibrinolysis and proteolysis [17,18]. Insulin resistant states such as obesity and T2DM are known prothrombotic states characterized by elevated PAI-1 levels [19]. In the Insulin Resistance Atherosclerosis Study, plasma C-reactive protein and PAI-1 levels were enhanced in insulin-resistant subjects who later developed diabetes, and PAI-1 levels predicted diabetes independently of other known risk factors [20]. However, the role of plasma FFA in thrombogenesis in humans is poorly established and no strong direct evidence is available.

Myeloperoxidase (MPO) is an enzyme derived from granules in activated neutrophils, monocytes and some tissue macropahges that catalyzes the formation of a number of reactive oxidant species (ROS) by the generation of chlorinating, nitrating, and other oxidizing species [21]. These products may initiate lipid peroxidation and promote post-translational modification of target proteins as part of the innate immune response. MPO and its reactive oxygen species (ROS) are enriched in human atheroma plaques [22-26] and increase within the area of infarct after an acute myocardial infarction [24,27]. Increased plasma levels of MPO independently predict endothelial dysfunction and coronary artery disease (CAD) [21], even after adjusting for traditional risk factors or hsCRP. In subjects presenting with acute coronary events, serum MPO levels is a strong predictor of adverse cardiac outcomes [28-30]. Circulating MPO concentrations also predict future CAD in otherwise healthy individuals [31]. Finally, decreased plasma levels of MPO secondary to specific MPO polymorphisms appear to be cardioprotective in humans [28,32-35]. Taken together, the available evidence highlights the importance of MPO to cardiovascular disease although the factors modulating its activity in humans remain poorly understood.

There is an increasing awareness about the potential role for FFA in atherosclerosis [36,37], although this area has been relatively neglected in the field. It has been noted that FFA may increase the production of multiple cytokines by mononuclear cells with generation of ROS and activation of pro-inflammatory NF-κB pathways in human endothelial cells [8]. Pharmacologic increases of plasma FFA (i.e., 5-fold elevation) by lipid infusion cause endothelial dysfunction and may alter plasma sCAM concentrations in healthy subjects [38,39], but the clinical relevance is not clear because these studies increased plasma FFA well beyond the physiological range.

With the widespread epidemic of obesity and diabetes, we carried out a proof-of-concept study to understand the role of elevated plasma FFA in relation to endothelial activation, vascular inflammation, MPO expression and the promotion of a prothrombotic state. The role of FFA on early steps of atherogenesis could have far reaching implications regarding the prevention and treatment of cardiovascular in obesity and T2DM.

## Research Design & Methods

### Subjects

Forty subjects participated in the study. Their clinical and laboratory characteristics are shown in Table 1. All subjects had a normal 75-gram oral glucose tolerance test (OGTT) performed at our clinical research unit. Physical activity was avoided in the days prior to testing or between study admissions. Body weight and degree of physical activity were stable in all subjects for at least 3 months prior to enrolment. No subjects had any evidence of cardiac, hepatic, renal or any other organ system disease, as determined by a complete medical history, physical examination, electrocardiogram, routine blood work, and urinalysis. No participants were receiving any medications known to affect carbohydrate metabolism. Tobacco users were excluded from participation because smoking alters insulin sensitivity and endothelial function. Each subject gave written informed consent before participation. The study protocol was approved by the Institutional Review Board of the University of Texas Health Science Center at San Antonio, Texas.

**Table 1:** Patient Characteristics

N (male/female)	40 (19/21)
Age (years)	38 ± 3

BMI (kg/m^2^)	28 ± 1

Fasting plasma glucose (mg/dl)	95 ± 1

2-hr plasma glucose (mg/dl)	120 ± 3

HbA_1c _(%)	5.3 ± 0.1

Fasting plasma insulin (μU/ml)	9 ± 1

2-hr insulin (μU/ml)	47 ± 7

Fasting plasma FFA (μU/ml)	544 ± 31

2-hr plasma FFA (μU/ml)	122 ± 7

Triglycerides (mg/dl)	104 ± 8

HDL-cholesterol (mg/dl)	44 ± 2

Systolic blood pressure (mmHg)	124 ± 3

Diastolic blood pressure (mmHg)	72 ± 2

### Experimental design

After the initial screening visit, all subjects were admitted to the research unit at 0700 h on day 1, following a 12-hour fast. Subjects were admitted twice 2-4 weeks apart, for the infusion in random order of normal saline or lipid (Liposyn III, a 20% triglyceride emulsion largely composed of soybean oil). Lipid or saline were infused at a constant rate of 0.5 ml/min (30 ml/hour) during the entire 2-day admission through an antecubital forearm vein. The lipid infusion was set to achieve a target day-long plasma free fatty acid (FFA) concentration of ~600 μmol/l, similar to that of subjects who are obese or have T2DM. Participants received a weight-maintaining diet prepared by the research dietician, consisting of 50% carbohydrate, 30% fat, and 20% protein. Meals were given at 0800 h, 1200 h, 1800 h, and 2100 h with a caloric distribution of 30%, 30%, 30% and 10% of total daily calories in each meal, respectively. Subjects consumed identical meals during each hospital admission. Complete food intake was confirmed after each meal by a research nurse. During days 1 and 2, blood was drawn every 2 hours from 0800 h through midnight and overnight every 4 hours for the determination of plasma glucose, C-peptide, insulin and FFA concentrations. On day 3, starting at 0700 h patients underwent a 2-hour hyperglycemic clamp. Blood was drawn for the measurement of serum ICAM-1, VCAM-1, MPO, E-selectin, tPAI-1 fasting in 2 separate samples 10 minutes apart before the start of the hyperglycemic clamp. Patients were discharged after completion of the hyperglycaemic clamp test. All procedures were performed in an identical fashion in both admissions.

### Hyperglycemic clamp

On day 3 and after an overnight fast, subjects underwent a hyperglycemic clamp as described previously by our group [40,41] to assess insulin sensitivity as the metabolic clearance of glucose (M) divided by the plasma insulin concentration (I) (or M/I). In brief, a 20-gauge Teflon catheter was inserted into an antecubital vein at 0800 h for the infusion of 20% dextrose. A second vein on the dorsum of the hand is cannulated retrogradely for the collection of blood samples, and the hand placed in a thermoregulated box at 65°C to achieve arterialization of the venous blood. Both intravenous lines are kept patent with a slow infusion of normal saline. After the collection of baseline samples, plasma glucose concentration is acutely raised by 125 mg/dL above the basal level and the desired hyperglycemic level is maintained (± 5%) for the following 120 min by periodic adjustment of a 20% glucose infusion based upon the negative feedback principle.

### Analytical determinations

The plasma glucose concentration was determined in duplicate by the glucose oxidase method with a Beckman Glucose Analyzer II (Beckman Instruments Inc, Fullerton, CA). Plasma insulin and C-peptide concentrations (Coat-A-Count Insulin, Diagnostic Products Corp., Los Angeles, CA) were determined by radioimmunoassay. Plasma FFA concentration was measured by standard colorimetric methods. Plasma ICAM-1, VCAM-1, MPO, E-selectin and tPAI-1 concentrations were assayed by enzyme linked immunosorbent assay (Lincoplex assay, Millipore Corp., MA).

### Statistical analysis

All values presented as the mean ± standard error of the mean. Within-group differences were determined by the paired two-tailed Student's *t *test. Normal distribution was checked before all analyses, and nonparametric estimates were used when appropriate. Comparisons were considered statistically significant if the *P *value was < 0.05. Where appropriate regressions were calculated by least squares linear correlation coefficients analysis. Analysis were performed using JMP software for Macintosh (SAS institute INC, Cary, NC).

## Results

### Plasma glucose, FFA and hormone concentrations during the 48-hour saline and lipid infusion

The plasma glucose, FFA, C-peptide and insulin concentrations during the 48-hour saline or lipid infusions are shown in Table 2. Mean 48-hour plasma FFA concentration increased significantly during the lipid infusion from 422 ± 80 to 588 ± 111 μmol/L (p < 0.001). There was a small but significant increase in mean 48-hour plasma glucose during lipid infusion compared to saline infusion (94 ± 18 to 97 ± 18 mg/dl, p < 0.02). This was likely a consequence of FFA-induced insulin resistance as evidenced by the increase in the mean plasma insulin and C-peptide concentration during the 48-hour lipid infusion (p = 0.01 and p = 0.04, respectively; Table 2).

**Table 2:** Effect of Lipid Infusion on Metabolic Parameters

48-hour mean values	Saline	Lipid
Glucose (mg/dl)	94 ± 18	97 ± 18*

FFA (μmol/L)	422 ± 80	588 ± 111**

Insulin (μU/ml)	8 ± 1	12 ± 2^†^

C-peptide (ng/ml)	3.3 ± 0.6	3.9 ± 0.7^††^

*p = 0.02; **p < 0.001; ^†^p = 0.01; ^††^p = 0.04

### Effect of a 48-hour increase low-dose lipid infusion on insulin sensitivity (Figure 1)

**Figure 1:**
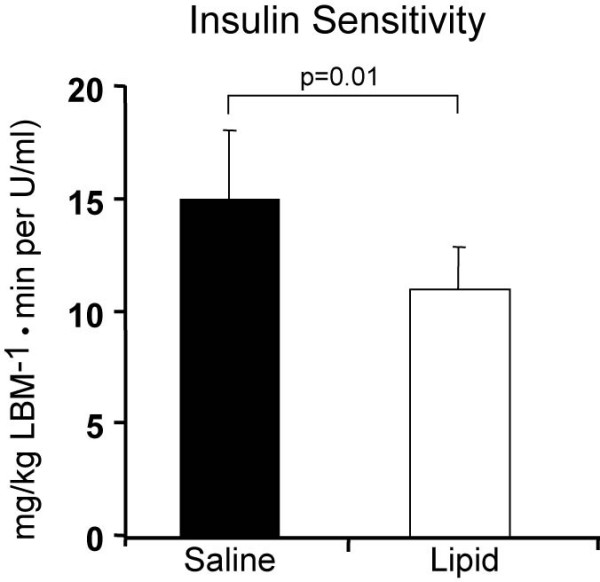
Effect of a 48-hour increase low-dose lipid infusion on insulin sensitivity

Lipid infusion significantly decreased insulin sensitivity as shown in Figure 1 with a 27 ± 4% reduction as measured by the M/I index (p = 0.01). This observation made evident that a mild physiological increase in plasma FFA by a short-term (48-hours) low-dose lipid infusion is capable of profound metabolic effects in healthy humans, consistent with prior observations by our group [41,42].

### Effect of a 48-hour increase low-dose lipid infusion on plasma concentrations of markers of endothelial activation, MPO and tPAI-1

**Figure 2:**
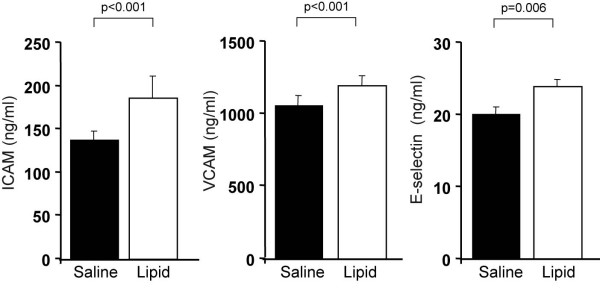
Effect of a 48-hour increase low-dose lipid infusion on plasma concentrations of markers of endothelial activation, MPO and tPAI-1

Compared to a 48-hour saline infusion, lipid infusion led to increased plasma ICAM-1 by 35 ± 5% (from 138 ± 10 vs. 186 ± 25 ng/ml), VCAM-1 by 13 ± 3% (1066 ± 67 vs. 1204 ± 65 ng/ml, both p < 0.001) and E-selectin by 17 ± 1% (20 ± 1 vs. 24 ± 1 ng/ml, p = 0.006) levels (Figure 2). The mean plasma FFA levels achieved with lipid infusion correlated closely with all plasma endothelial activation markers: ICAM (r = 0.38, p = 0.03), VCAM-1 (r = 0.48, p < 0.01) and E-selectin (r = 0.48, p < 0.01).

**Figure 3:**
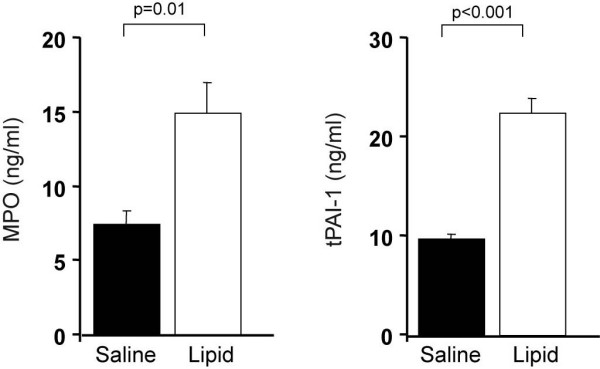
Compared to a saline, FFA increased doubled plasma MPO from 7.5 ± 0.9 to 15 ± 25 ng/ml (p = 0.01) and tPAI-1 by 132% from 9.7 ± 0.6 to 22.5 ± 1.5 ng/ml (p < 0.001)

**Figure 4:**
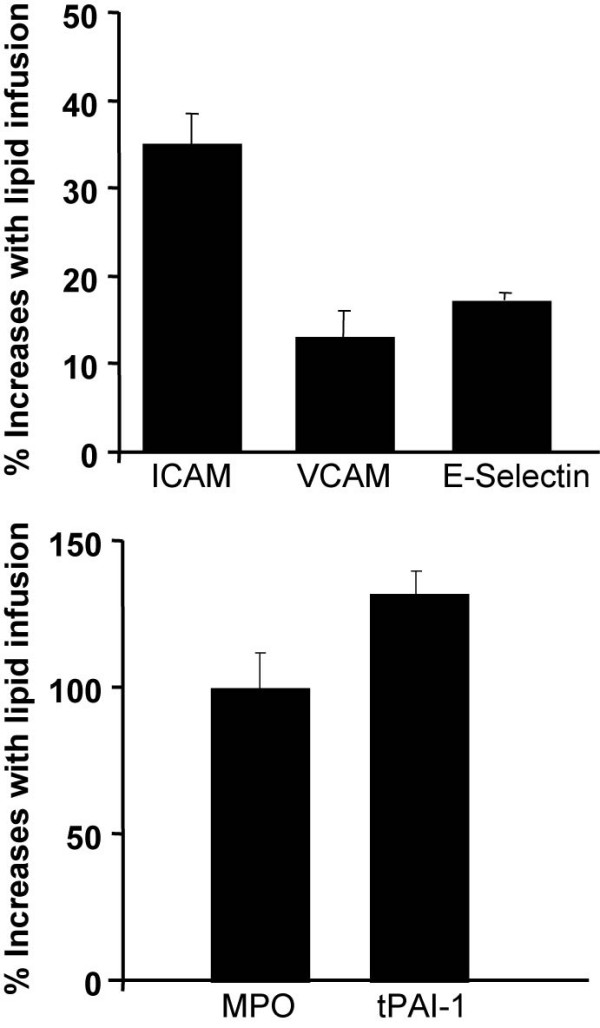
This figure summarizes the percent increase with lipid infusion of markers of endothelial activation, MPO and tPAI-1

Plasma MPO and tPAI-1 levels were also altered by FFA elevation and to a greater extent. Compared to a saline, FFA increased doubled plasma MPO from 7.5 ± 0.9 to 15 ± 25 ng/ml (p = 0.01) and tPAI-1 by 132% from 9.7 ± 0.6 to 22.5 ± 1.5 ng/ml (p < 0.001) (Figure 3). Figure 4 summarizes the percent increase with lipid infusion of markers of endothelial activation, MPO and tPAI-1. The increase in plasma FFA achieved with lipid correlated very strongly (r = 0.69, p < 0.001) with the increase in plasma tPAI-1, suggesting a close relationship between FFA and induction of a prothrombotic state under these experimental conditions.

**Figure 5:**
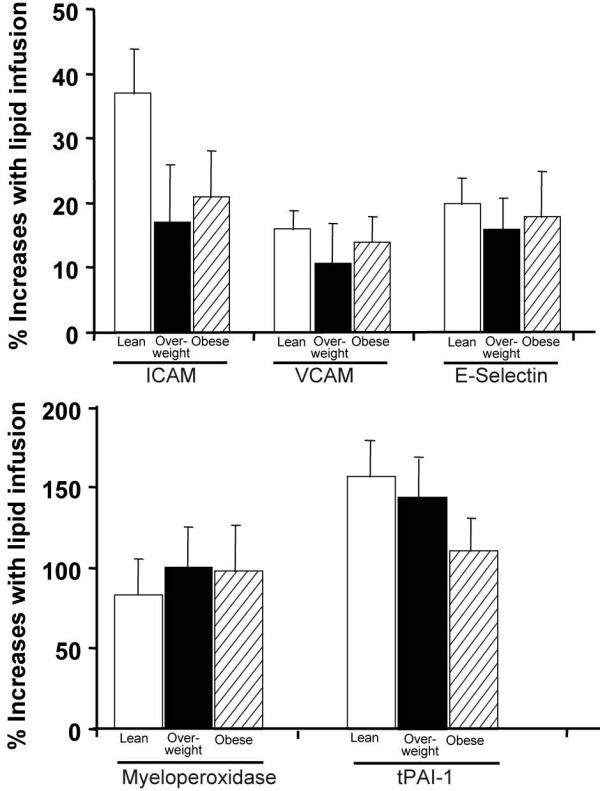
**Figure**5**describes the response in subjects divided by BMI as either lean (BMI <25 kg/m^2^), overweight (BMI >25 and <30 kg/m^2^) or obese (BMI >30 kg/m^2^)**

Finally, we explored if total body fat could modify or play a role in the marked increase in sCAM, MPO or tPAI-1 response to 48-hour FFA stimulation. Figure 5 describes the response in subjects divided by BMI as either lean (BMI <25 kg/m^2^), overweight (BMI >25 and <30 kg/m^2^) or obese (BMI >30 kg/m^2^). No significant differences were appreciated for any variable based on BMI, suggestive of a direct effect of FFA-induced endothelial activation independent of total adiposity.

## Discussion

Few clinical studies have examined the role of FFA as a trigger for endothelial activation, inflammation and thrombosis. This has been overlooked in favour of a focus on traditional cardiovascular risk factors or detailed studies on lipoprotein metabolism. With obesity and T2DM reaching epidemic proportions, it is important to assess the role of excessive FFA supply regarding endothelial injury and inflammation because both conditions are characterized by increased rates of lipolysis and plasma FFA due to adipose tissue insulin resistance. In order to have clinical relevance, the study carefully mimicked the plasma FFA levels characteristic of obese and diabetic patients and assessed their impact by using validated plasma markers of endothelial activation, systemic inflammation and thrombosis.

Serum cellular adhesion molecule levels increase in association with cardiovascular risk factors and are associated with structural functional measures of atherosclerotic disease, as well as with adverse cardiovascular prognosis [9,11,15,43,44]. Serum VCAM-1, ICAM-1 and E-selectin concentrations are elevated in obesity [45-47], chronic renal failure [48], in lean and obese subjects genetically predisposed to T2DM [39,49] and in T2DM [16,50]. Recently, ICAM-1 and E-selectin were reported to predict future development of T2DM, even after accounting for classical risk factors such as age, BMI, family history of T2DM, hsCRP and others [51]. Taken together, these studies are an indication of the value of elevated plasma VCAM-1, ICAM-1 and E-selectin levels to assess early systemic inflammation and EC activation. Elevated plasma FFA offer a unifying mechanism as a cause not only for the development of insulin resistance, as reported in the literature (reviewed by Cusi in [52]) and observed in this study, but as a factor actively involved in the higher cardiovascular risk of obese and insulin-resistant populations. The results of this study also highlight the susceptibility of ECs to modest increases in plasma FFA, as endothelial activation was induced with just a 2-day low-dose lipid infusion. However, it must be recognized that future studies should examine the role of FFA using gold-standard techniques to assess endothelial function [11] and evaluate their long-term effect on the vascular bed. Finally, because the magnitude of the elevation of sCAM was independent of adiposity and pre-existing insulin resistance (i.e, overweight and obese vs. lean subjects; Figure. 5), this renders further support for the hypothesis of a direct effect of plasma FFA elevation to induce markers of endothelial activation and vascular inflammation. Indeed, there was a strong correlation between the plasma FFA level achieved by lipid infusion and the elevation on biomarkers of EC activation.

The novel finding that a mild elevation in plasma FFA may activate vascular MPO and tPAI-1 has important clinical implications. The mechanisms by which MPO may promote atherogenesis include conversion of LDL into more atherogenic oxidized particles (oxLDL), oxidative modification of apolipoprotein A-I that results in a dysfunctional HDL and reduction of EC nitric oxide availability resulting in endothelial dysfunction [21-24,26,53-55]. These multiple mechanisms help explain the strong predictive value of plasma MPO levels for acute coronary syndromes (ACS) in humans even after adjusting for traditional cardiovascular risk factors, Framingham risk score, or hsCRP [28-30]. For instance, Zhang et al [28] in a case-control study in a tertiary care referral center, compared 158 patients with documented CAD against 175 patients without angiographically significant CAD (controls) and found that both leukocyte- and blood-MPO levels were significantly greater in patients with CAD with an odds ratio (OR) of 20.4 (95% CI, 8.9-47.2) for the highest vs. lowest quartiles of plasma MPO levels. Brennan et al [29] studied 604 patients who presented at the emergency room with ACS and reported that those with the highest MPO quartile has a 3.9-fold higher risk of having a CHD event and an even higher predictive value in the next 6 months. Similar results have been reported by Baldus et al [30]. Plasma MPO has been accepted to be a good biomarker of endothelial dysfunction [56] and predicted cardiovascular events even in 1,138 apparently healthy subjects in the EPIC-Norfolk Prospective population study [31]. MPO-triggered EC apoptosis, intracoronary erosions and thrombus formation has been proposed based on work by Sugiyama, Libby et al [24]. This link may be further strengthened by this report and may point to elevated FFA as a common pathogenic mechanism for endothelial dysfunction, inflammation and thrombogenesis.

Several mechanisms may explain how lipid infusion may induce endothelial activation and eventual damage. Elevation of plasma FFA by lipid infusion activates pro-inflammatory genes such as TNF-α, which is a potent stimulator of sCAMs and MPO secretion [8,36,37]. *In vitro *studies in endothelial and vascular smooth muscle cells have provided evidence that FFA increase oxidative stress and inflammation by activating the NF-κB pathway and increasing the formation of ROS by mononuclear cells, which initiate the inflammatory process involving the endothelium. Recent studies indicate that there is a clear fatty acid dose-response impairment of insulin signalling, inhibition of nitric oxide production and activation of NF-κB activity in bovine aortic endothelial cells [57] and in mononuclear cells of healthy subjects exposed to acute pharmacological increases in plasma FFA [38]. Human monocytes exposed for just 48 hours to excessive lipids have a dose-dependent increase in intercellular ROS and increased adhesion to ECs, mediated by an increase in integrin CD11b cell surface expression [58]. In healthy subjects, fatty acid-induced oxidative stress and endothelial activation with an increase in plasma TNF-α, IL-6, ICAM-1 and VCAM-1 can be induced by a single high-fat meal [59]. Endothelial dysfunction has been reported to be reversible with lifestyle changes [60], anti-inflammatory agents such as salicylates [61] or by insulin-sensitizers such as thiazolidinediones [62]. Thus, increased lipid infusion and/or plasma FFA appears to be an early trigger for multiple pathways leading to atherogenesis, independent of FFA-induction of muscle or liver insulin resistance. A limitation of the study is that there is no commercially available mixture that mimics the human fatty acid profile. We used Lypsoyn III that is 100% soybean oil, which is composed of largely unsaturated long chain fatty acids, with 55% linoleate, 22% oleate, 11% palmitate and 4% stearate while plasma is higher in more saturated fatty acids with 11% linoleate, 38% oleate, 28% palmitate and 12% stearate [63]. The overall impact of different fatty acids on the vascular bed has not been carefully characterized in humans but in skeletal muscle palmitate may induce insulin resistance to a greater extent than other fatty acids [64]. However, this has not been confirmed in others studies in which linoleate, oleate and palmitate had similar inhibitory effects on glycogen synthesis and insulin-stimulated muscle glucose uptake [65,66]. Clearly more work is needed in this field but unfortunately plasma FFA have been poorly studied and overall neglected as relevant in the pathogenesis of atherosclerosis in humans. We believe that this proof-of concept study may be a provocative and valuable contribution to stimulate future work in this area.

## Conclusions

We have demonstrated that a sustained low-dose lipid infusion leading to a modest increase in plasma FFA concentration is sufficient to induce endothelial activation, increase plasma myeloperoxdase levels and promote a prothrombotic state in non-diabetic healthy subjects. Taken together, these results provide direct evidence in humans that mild short-term lipid-oversupply is sufficient to initiate early vascular abnormalities that may lead to atherosclerosis and CVD.

## Competing interests

The authors declare that they have no competing interests.

## Authors' contributions

KC conceived the study, designed and performed the study experiments, analyzed the data and interpreted the results and wrote the manuscript. ET and MM performed the study experiments, interpreted the results, participated in the writing and critically revised the manuscript. All authors read and approved the final manuscript.
